# Cerebral blood perfusion changes in amputees with myoelectric hands after rehabilitation: a SPECT computer-aided analysis

**DOI:** 10.1186/s12868-016-0294-3

**Published:** 2016-08-31

**Authors:** Qiufang Liu, Xiujuan Zheng, Panli Li, Lian Xu, Longwen He, Zhao Mei, Yinyan Zhu, Gang Huang, Chunlong Zhong, Shaoli Song

**Affiliations:** 1 Department of Nuclear Medicine, Ren Ji Hospital, School of Medicine, Shanghai Jiao Tong University, 160 Pujian Road, Shanghai, 200127 China; 2 Department of Automation, School of Electrical Engineering Information, Sichuan University, Sichuan, 610065 China; 3 Shanghai Rehabilitation and Vocational Training Center for the Disabled, Shanghai, 200127 China; 4 Department of Neurosurgery, Ren Ji Hospital, School of Medicine, Shanghai Jiao Tong University, 160 Pujian Road, Shanghai, 200127 China

**Keywords:** Cerebral blood flow, SPECT/CT, Computed-aided method, Myoelectric hand, Rehabilitation

## Abstract

**Background:**

Rehabilitation, which is essential for amputees with myoelectric hands, can improve the quality of daily life by remodeling the neuron network. In our study, we aim to develop a cerebral blood perfusion (CBF) single-photon emission computed tomography computer-aided (SPECT-CA) detection scheme to automatically locate the brain’s activated regions after rehabilitation.

**Results:**

Five participants without forearms (three male, two female, mean age 51 ± 12.89 years, two missing the right side, and three missing the left side) were included in our study. In the clinical assessment, all of the participants received higher scores after training. The results of the SPM analysis indicated that CBF in the precentral gyrus, postcentral gyrus, frontal lobe, temporal lobe and cerebellum was significantly different among the five participants (*P* < 0.05). Moreover, SPECT-CA showed that the activated brain areas mainly included the precentral gyrus, postcentral gyrus, cerebellum and extensive cerebral cortex.

**Conclusion:**

Our study demonstrated that the CBF SPECT-CA method can detect the brain blood perfusion changes induced by rehabilitation with high sensitivity and accuracy. This method has great potential for locating the remodeled neuron regions of amputees with myoelectric hands after rehabilitation.

**Electronic supplementary material:**

The online version of this article (doi:10.1186/s12868-016-0294-3) contains supplementary material, which is available to authorized users.

## Background

It is projected that the number of people living with the loss of a limb in the United States will increase to 3.6 million by the year 2050 [1]. As a result, amputees face a number of problems in daily life. Myoelectric hands can partially compensate for functions of the hand according to the amputee’s will. Amputees can control myoelectric hands by voluntarily contracting their residual muscles, which can generate myoelectric signals and are received by two cutaneous electrodes attached to the residual muscles [2]. Due to the changes in the electrical signal of the muscles, the amputees can drive a set of motors in the myoelectric hand within the prosthetic device, allowing them to open and close or grasp and pinch. In addition, the enhanced utility the myoelectric hand of amputees was found to have a beneficial effect on both phantom limb pain and cortical reorganization [3, 4]. Therefore, this device is widely applied in amputees.

Rehabilitation is required before amputees can fully use their device. During rehabilitation, amputees can learn how to control their residual muscles to reach threshold levels of myoelectric signal strength that can induce the motion of the myoelectric hand. At the same time, rehabilitation may induce neuron network remodeling. Previously, it has been confirmed that the microstructure of the brain is not fixed in adulthood but rather is susceptible to experience-dependent structural plasticity throughout life [5]. Furthermore, studies have indicated that training can induced gray matter changes [6, 7]. The association between rehabilitation, brain activation and performance in controlling of the myoelectric hand is recognized important but has seldom been explored.

Brain imaging, such as functional magnetic resonance (fMRI) and CBF SPECT/CT, can provide useful brain functional information. Additionally, the computer-aided method is used for the quantitative analysis and detecting target regions with higher sensitivity and accuracy [8, 9]. Several studies have focused on the changes during manipulating the prosthesis and described activation regions in fMRI examinations [10, 11]. CBF SPECT/CT imaging is used to measure regional cerebral blood flow changes elicited by stimulation or activation of behavioral functions, which may reflect changes in the underlying neuronal network. However, there has been little research using the SPECT-CA method on the brain perfusion changes of amputees after rehabilitation with myoelectric hands.

In this study, we conducted CBF SPECT/CT imaging before and after rehabilitation. Subtracted images from the two time points for each participant were analyzed by our computer program to automatically detect and quantify the activated regions. The purpose of this study was to develop a CBF SPECT-CA detection scheme to automatically locate the activated brain regions that were associated with rehabilitation and to study the association between rehabilitation, brain activation and the ability to control the myoelectric hand.

## Methods

### Participants

Five participants without forearms (three male, two female, mean age 51 ± 12.89 years, two missing the right side and three missing the left side) were studied. Subjects were eligible for inclusion if they were without motor problems or neurologic or psychiatric history, had normal or corrected-to-normal sight and had no earlier experience with a prosthetic or simulator. The study was approved by the Ethics Committee of Ren Ji Hospital, and informed consent was provided by all subjects before participation.

### Rehabilitation

Rehabilitation was provided for 8 weeks and included basic myoelectric signal training, feedback training and activities of daily living (ADL) training [12, 13].

Basic myoelectric signal training was used to teach the participants how to activate and isolate their individual signals. It consisted of three parts and lasted for 2 weeks: imaginary training, muscle-strength exercise and electromyography (EMG) strength training. In part 1, participants were asked to complete a variety of actions, such as opening and closing the hand with an imaginary hand. Each exercise was performed in three groups, 80 times per group, and limited to 3 min. In part 2, participants used the self-made up-limber muscle training device, which weighed 4 kilograms and was hung onto the residual stump, to perform elbow flexion and extension as well as shoulder adduction and abduction. The training was given in three groups, 30 times per group at 2-min intervals. In part 3, the EMG signals were measured by two EMG electrodes that were placed separately on the extensor carpi ulnaris and flexor carpi ulnaris and linked to an MT-2 EMG trainer (Kesheng prostheses corporation, Shanghai, China). The participants attempted to enhance the EMG signal by controlling muscle strength, reach a signal strength reach 40 μV or above and maintain this signal for 2 s. In this part, we adjusted the position of the electrodes and gradually found the spot where the signal was the maximum. This process was completed in six groups total, with each EMG electrode corresponding to three groups, 30 times per group.

In the process of feedback training, we used computer software to provide visual feedback to the participants where the movement of a three-dimensional (3D) virtual prosthetic hand was controlled by the participants’ muscles. EMG electrodes were placed on the best spot, and the signal was generated by the participants controlling their muscles and was passed to the software matched to the EMG trainer in the computer. Participants were instructed to open and close the hand fully and continuously. The training was completed in four groups per day for 1 h per group. After opening and closing the hand 20 times, participants would take a short break to avoid muscles fatigue. The feedback training lasted for 2 weeks.

Daily life training helped the participants learn how to perform activities of daily living with actual myoelectric prostheses. ADL training mainly included (1) grasping and moving a water glass, (2) taking a weight, (3) opening and closing a door and (4) carrying books and newspapers. The training was performed in three groups per day, 20 times per group. The ADL training lasted for 4 weeks.

### Image acquisition

CBF SPECT/CT imaging was performed before and after 8-weeks of rehabilitation using ^99m^Tc-ECD (Shanghai GMS Pharmaceutical Corporation, Shanghai, China). The radiopharmaceutical was injected intravenously at a dose of 1110 MBq in a quiet environment. Patients were in a supine position and were instructed to remain calm with their eyes closed. SPECT acquisition was performed for 20–30 min after injection, using a rotating, double-head, large-field-of-view gamma camera (PHILIPS Precedence, Philip Medical Systems, Netherlands), equipped with low-energy, high-resolution collimators. The data were collected in a 128 × 128 matrix through a 360° rotation at 6° intervals. The CT scan was performed using a multi-slice CT Scanner (PHILIPS Precedence, Philip Medical Systems, Netherlands, 140 kv, 35 mAs, 3 mm per scan). Reconstruction of transaxial slices was performed by filtered back projection (Metz filter power, 3.00; full width at half maximum, 10 mm) with subsequent attenuation correction using the Chang method (attenuation coefficient of 0.12).

### Evaluation

Evaluation was performed before and after the 8 weeks of rehabilitation. In the evaluation process, participants with myoelectric hands underwent the myoelectric signal assessment, brief Fugl-Meyer Motor Function assessment, ADL assessment and balance test. Brief Fugl-Meyer Motor Function and ADL assessments were performed according to the supplemental materials (Additional file 1). In the myoelectric signal assessment, we recorded the threshold signal intensity of each participant, which is the minimum signal that can induce the movement of myoelectric prosthetic hands. We defined the EMG signal that exceeded the threshold signal as an effective EMG signal. The frequencies and duration of effective EMG signals were used as the index when assessing the myoelectric signal in those participants. The stability index (ST) was assessed in the Tetrax balance test system (Tetrax, Sunlight, Israel) to evaluate the balance function. The participants stood on the center of the sensor with their hands naturally hanging down or flat, with their eyes open or closed and while standing on one foot or two feet. By measuring and analyzing the mechanical signal changes of vertical pressure on the feet, the Tetrax balance test system yielded the ST, which can reflect the overall stability of the participant. With the ST, a higher value represents a worse balance function.

### SPECT-CA method

To detect the changes in all five participants after training, the SPM toolbox (SPM8, Wellcome Trust Centre for Neuroimaging, University College London, London, UK) and PickAtlas software (WFU Pickatlas, version 3.0) were used for analyzing the SPECT images [14, 15]. For each subject, the pre- and post-SPECT images were spatially normalized into the standard stereotactic Montreal Neurological Institute (MNI) space using a ^99m^Tc-ECD template and smoothed using a 12-mm isotropic Gaussian filter. Moreover, a general whole-brain mask was created based on an anatomical labeling atlas and adopted to all images for further statistical comparisons. Paired *t*-tests were performed to compare the pre-training and post-training group. A *p* value of less than 0.05 was considered statistically significant.

To validate the results of SPM analysis further, we also explored the changes in each participant using the SPECT-CA program. Pre- and post-CBF SPECT/CT imaging were performed to obtain baseline and follow-up SPECT images before and after rehabilitation for each participant. In our SPECT-CA program, the follow-up image was first aligned with the baseline image using a rigid registration algorithm for further comparisons. Then, the values were normalized for these two aligned images using the Z-map approach introduced by Boussio [16]. The change rate of the CBF was calculated voxel-by-voxel to generate a parametric image, noted as a change-rate map. Based on the change-rate map, the changed regions were automatically obtained using the K-means clustering method. Considering the SPECT image resolution and partial volume effects, the regions with higher average change rates (>25 %) and larger volumes (>200 voxels) were selected as activated regions according to our patent submitted to the state intellectual property office of the P.R.C [17]. According to the activated regions, the change rates of the regions are listed and reported with respect to the absolute pixel number, mean change rate and location for each participant.

## Results

### Clinical assessments

From the EMG signal assessment results, we found that after rehabilitation all of the participants could conduct more effective EMG signals for a longer duration ( Table 1). Moreover, all five participants received higher scores in the Fugl-Meyer Motor Function and ADL assessments after rehabilitation, consistent with the EMG signal assessment results (Table 1). The stability index declined, indicating that the participants’ balance function improved after rehabilitation. In addition, three of the participants (participants 1, 2 and 3) received a higher score on the assessment, which indicates that they could manipulate the myoelectric hand more accurately and consistently compared with the others.

**Table 1:** The clinical evaluation was performed before and after the rehabilitation. It contains four aspects: (1) EMG signal assessment (The frequency and duration of effective EMG signals was assessed in 60 s); (2) Fugl-Meyer Motor Function assessment (total scores = 34); (3) ADL assessment (total scores = 16); (4) Balance test

Participant	Frequencies (times)		Duration (second)		Fugl-meyer motor function scores		ADL scores		Stability index	
	Before	After	Before	After	Before	After	Before	After	Before	After
1	2	17	1	7	21	32	4	14	41	22
2	1	12	2	8	22	30	4	12	37	24
3	1	14	1	17	23	30	3	14	37	25
4	0	7	0	5	21	24	3	11	39	30
5	1	6	1	5	19	25	2	9	43	33

“Before” represents the assessment that was performed before rehabilitation and “After” represents the assessment that was performed after rehabilitation

### SPECT-CA analysis

The results of SPM analysis and paired *t*-tests indicated that it existed activated regions of all the 5 participants after training. And they were 5 regions had significant difference, which were precentral gyrus, postcentral gyrus, frontal lobe, temporal lobe and cerebellum (P < 0.05).

**Fig. 1:**
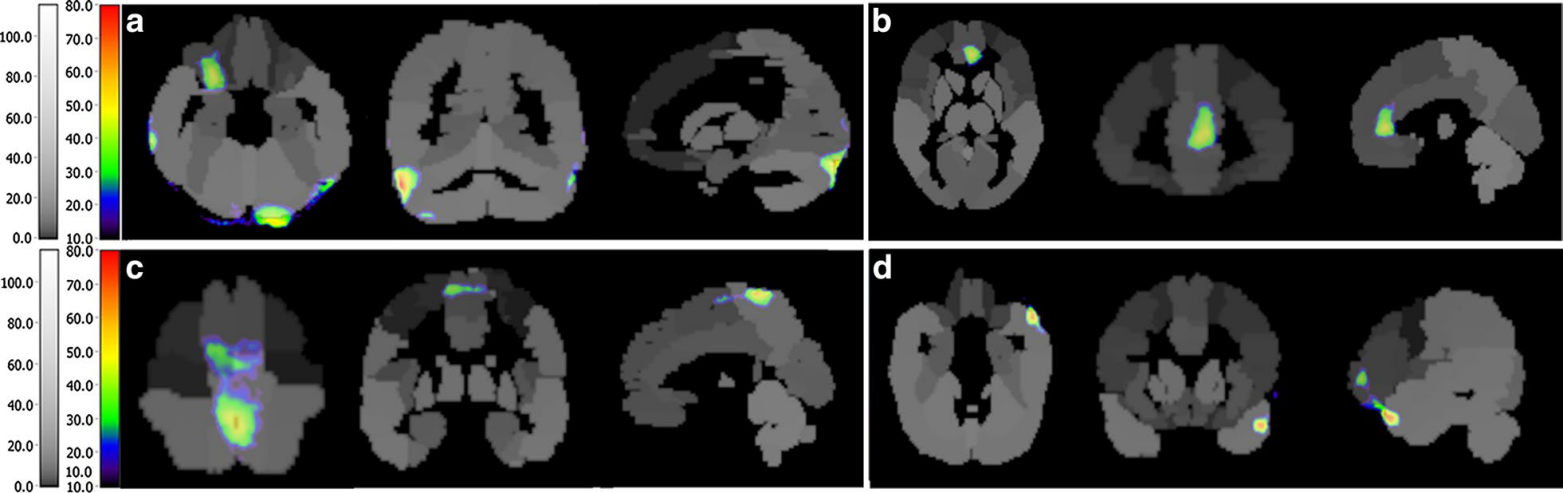
Activated regions of participant 1 after 8-weeks rehabilitation. **a** Cerebellum. **b** Frontal lobe. **c** Postcentral gyrus. **d** Temporal cortex

**Fig. 2:**
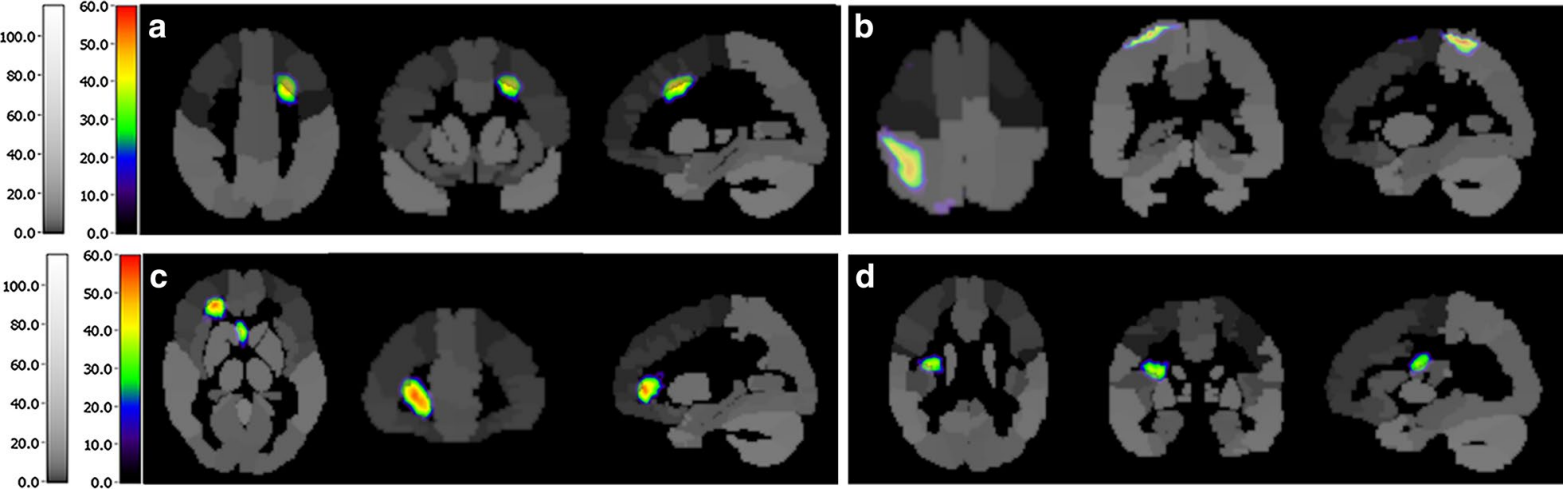
Activated regions of participant 2 after 8-weeks rehabilitation. **a** Precentral gyrus. **b** Postcentral gyrus. **c** Frontal lobe. **d** Opercula

**Fig. 3:**
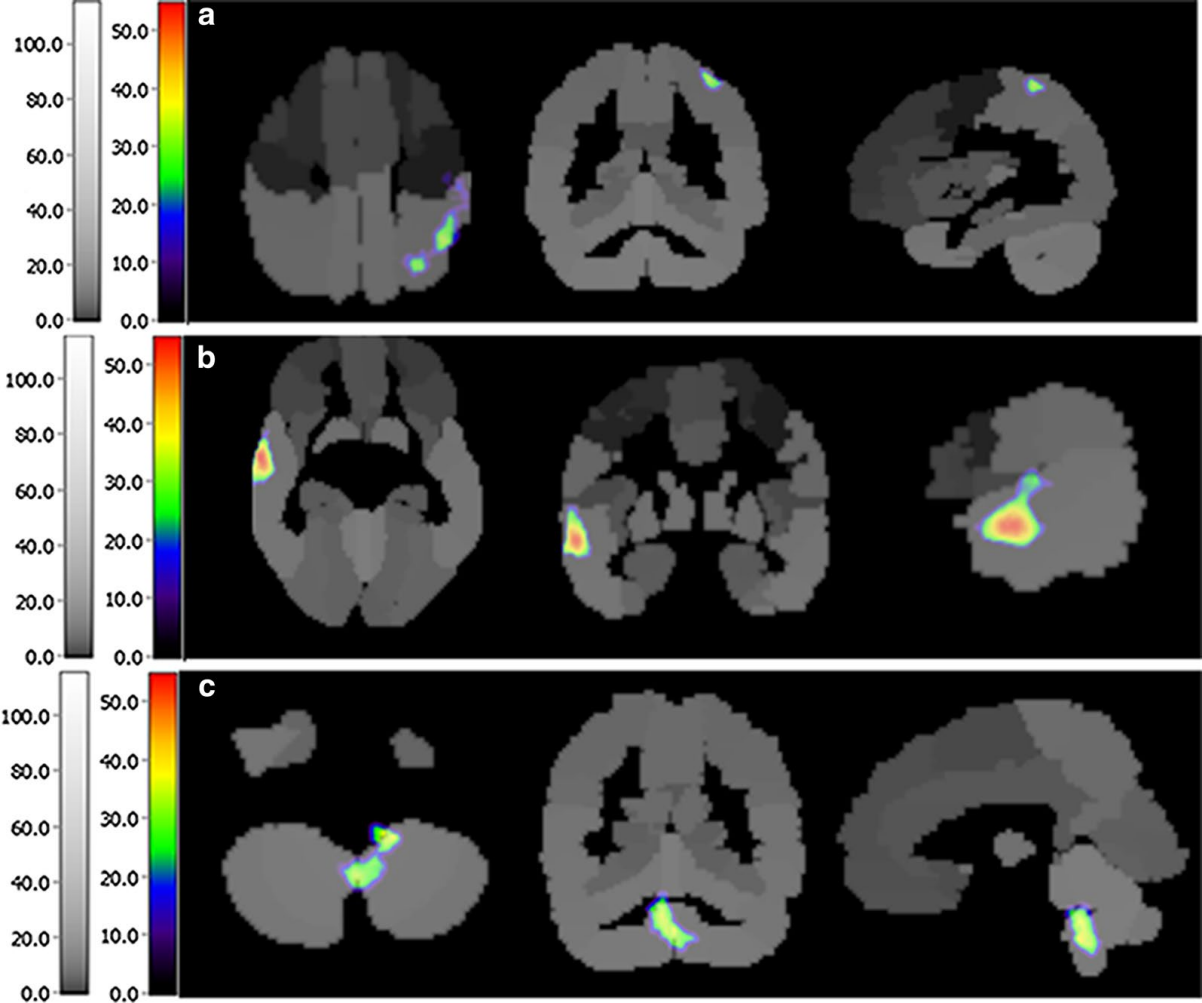
Activated regions of participant 3 after 8-weeks rehabilitation. **a** Postcentral gyrus. **b** Temporal cortex. **c** Cerebellum

**Fig. 4:**
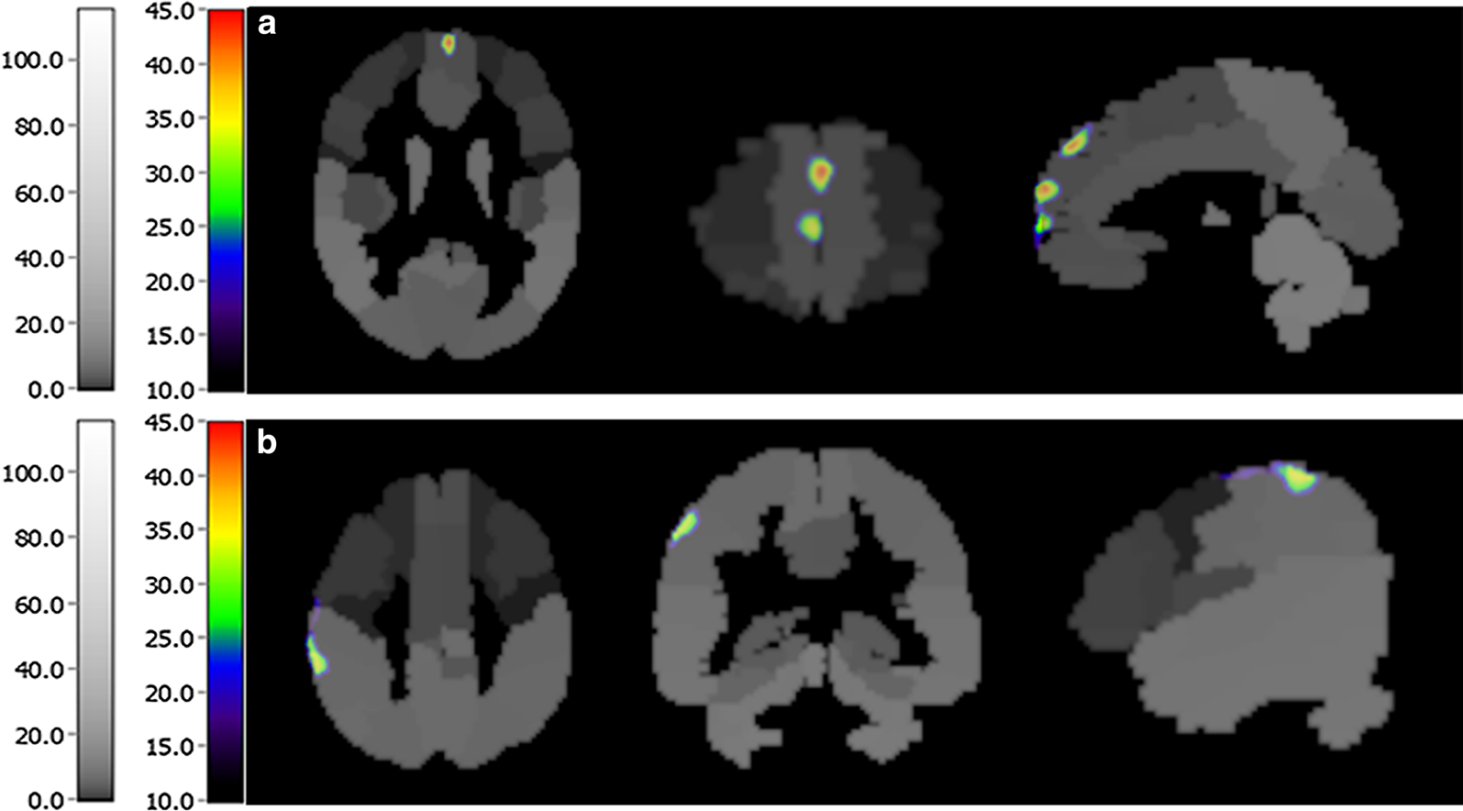
Activated regions of participant 4 after 8-weeks rehabilitation. **a** Frontal cortex. **b** Postcentral gyrus

**Fig. 5:**
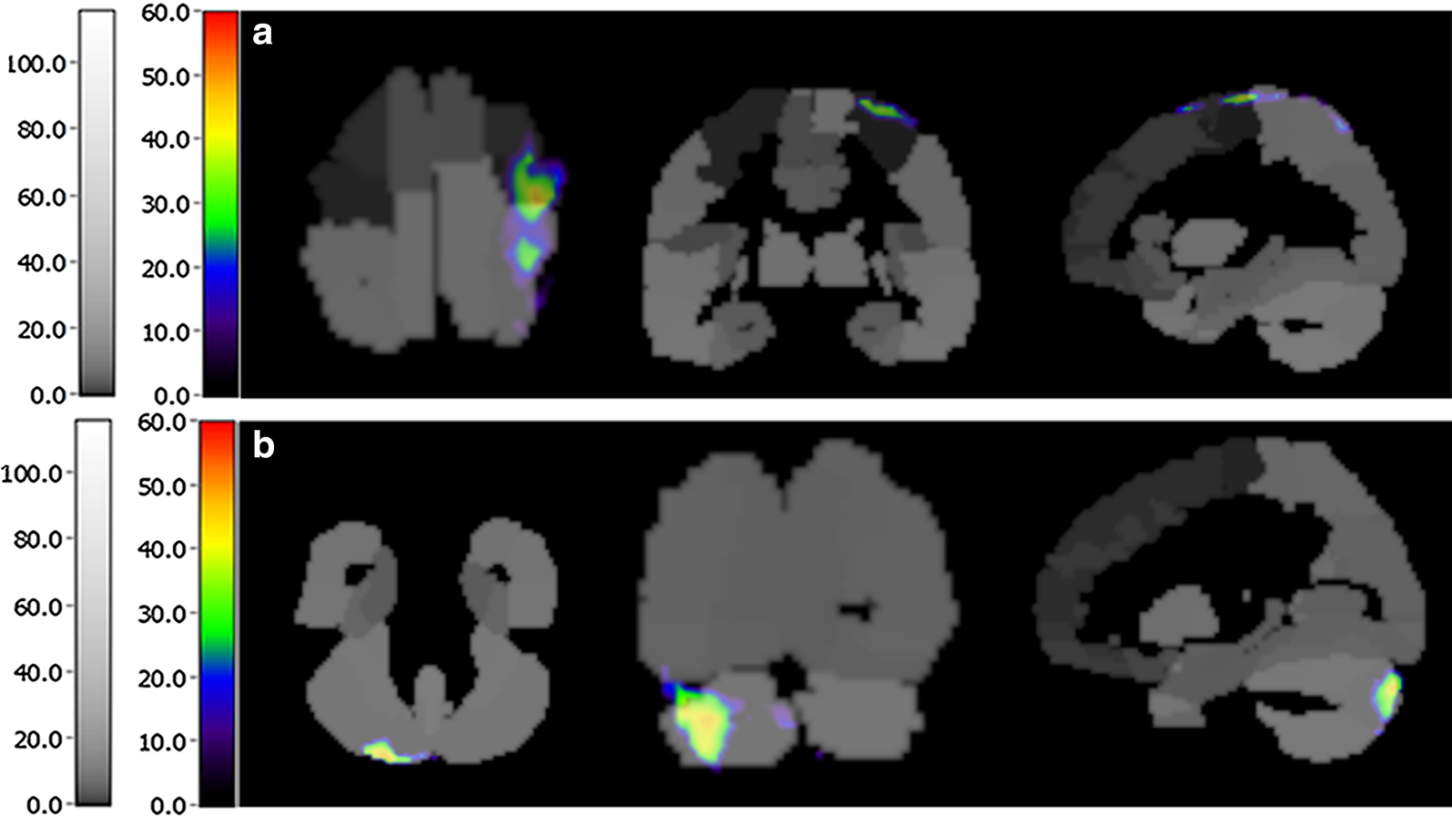
Activated regions of participant 5 after 8-weeks rehabilitation. **a** Precentral and postcentral gyrus. **b** Cerebellum

In addition, using our SPECT-CA program, we found that each participant displayed brain activation after rehabilitation, which was consistent with the SPM analysis. The results showed that participant 1 had seven brain activated regions, participant 2 had nine, and the remaining participants each had four activated regions (Table 2). Details of each active region are provided in the supplemental materials (Additional file 2). From the change-rate map, we found that the activated regions that were common between the five participants were mainly located in the precentral gyrus, postcentral gyrus and cerebellum. In addition, extensive areas of the cortex, including the temporal cortex, occipital cortex and frontal cortex, were activated. In Figs. 1, 2, 3, 4 and 5, the most important activated regions are illuminated using the AAL labeling brain atlas for each participant. The other details are provided in the supplementary files (Additional file 3).

**Table 2:** The details of the five participants and the results of SPECT-CA analysis

Participant	Age	Sex	Reason	Side	Mean ± SD of change-rate	Total activated regions	Relative activated regions
1	36	Male	Congenital malformation	Left	29.20 ± 0.8059	7	Postcentral gyrus, left temporal cortex, cerebellum, opercula cortex, fontal cortex
2	40	Male	Explosive wound	Right	27.90 ± 0.6423	9	Postcentral gyrus, precentral gyrus, opercula cortex, fontal cortex, cerebellum, temporal cortex
3	43	Female	Traffic accident	Left	30.20 ± 1.234	4	Postcentral gyrus, right temporal cortex, cerebellum, fontal cortex
4	65	Male	Traffic accident	Right	26.20 ± 0.4416	4	Postcentral gyrus, fontal cortex, cerebellum, occipital cortex
5	63	Female	Traffic accident	Left	26.53 ± 0.4732	4	Postcentral gyrus, cerebellum, frontal cortex

## Discussion

Our study showed that the precentral gyrus, postcentral gyrus, cerebellum and extensive cortex (temporal cortex, occipital cortex and frontal cortex) play a definitive role in regulating the movements and the perception of a prosthesis. As we know, the precentral gyrus is associated with motor commands, and the postcentral gyrus is related to the perception of movement; cerebellum activation was related to the regulation of body balance and muscle tension. Thus, rehabilitation appeared to increase local cerebral perfusion in areas that are involved with movement planning and execution, which might be a sign of an active cortical reorganization processes.

Many studies have been conducted on the plasticity of neural networks in response to injuries [18], training [11, 19], and treatments [20, 21] with imaging methods. The fMRI, PET and SPECT are the most commonly used techniques to evaluate the changes [22–24]. Blood oxygen level, regional cerebral blood flow or FDG uptakes are measured by these methods and can definitively reflect changes in the underlying neuronal activity. Our results corroborate with previous related articles. Maruishi et al. demonstrated that the right ventral premotor cortex and right posterior parietal cortex were involved in manipulating the EMG prosthetic hand [11]. Another study indicated that brain activation in Brodmann motor areas 6, 4, 3, 1 and 2; visual area 19; and the cerebellum were observed when the patient contracted the remaining stump muscles and thought about opening and closing her myoelectric prosthetic hand [25].

However, our results are not in full accord with theirs because in our study, extensive cortex, including the temporal cortex, occipital cortex and frontal cortex, was also activated. We attribute this to the fact that the participants in our study underwent 8 weeks of rehabilitation, in which they received complex motor skills that involve procedural learning. It is known that the frontal cortex is activated during response preparation and execution and that the temporal cortex and occipital cortex are associated with processing auditory and visual information, as well as being an integral part of the primary visual cortex output pathway, which has relevance with the process of spatial position and related motion control. Thus, the extensive cortex activated in our study is part of the visual-to-motor networks, which was conducted in our rehabilitation. Similar studies have also demonstrated increased gray matter density in the visual motion area, frontal and parietal cortex after as little as 7 days of short-term training [26, 27].

From the results, we found that participants 1, 2 and 3, who had more activated regions or higher change rates, had a better performance in the clinical assessments. This is because the more activated regions or higher change-rates indicate more neurogenesis, synaptogenesis, vascularization and myelination and axonal sprouting in the white matter [28, 29]. These are the basic components of the neural pathways to control the muscles and complete some movements. Furthermore, the amount and activity of the neurons is also responsible for the specification of movement parameters such as amplitude, continuity and speed of movement [30]. That is, the control of the speed, force and modulation of a myoelectric prosthesis depends on the activity of specific areas of the brain. Therefore, participants with more activated regions or higher change rates may have better control of the myoelectric prosthesis.

The CBF SPECT-CA program we used is automatic and can give fast, objective assessments of the whole brain. By analyzing the subtracted images of the baseline and follow-up SPECT imaging with this method, we could not only erase the effect of unequal injected activities of the images but also quantify the activated regions. More importantly, our results verify that it can detect the activated regions of amputees after rehabilitation with high sensitivity and accuracy. It has great potential for predicting the performance in controlling prostheses and locating the reconstruction of the neuron network induced by rehabilitation.

Furthermore, we found that there were differences among the five participants after rehabilitation, which may be due to different ages, residual muscles or amputation-specific reasons. Consequently, further research should recruit larger populations to validate the results and analyze the factors that may affect the results of rehabilitation and brain activation.

## Conclusion

In summary, our study demonstrated that the CBF SPECT-CA method can detect the brain changes induced by rehabilitation with high sensitivity and accuracy. This method is promising for locating the remodeling neuron regions of amputees with myoelectric hands after rehabilitation. In addition, participants with more activated regions or higher change rates may have better control of the myoelectric prosthesis.

## Additional files

10.1186/s12868-016-0294-3 The contents of Brief Fugl-Meyer Scale and ADL Assessment.
10.1186/s12868-016-0294-3 The change-rate of the 5 participants.
10.1186/s12868-016-0294-3 The activated regions of the five participants.

## Abbreviations

CBF: cerebral blood flow
SPECT-CA: single-photon emission computed tomography computered aided
^99m^Tc-ECD: ^99m^Tc-ethyl cysteinate dimmer
SPM: statistical parametric mapping
fMRI: functional magnetic resonance
ADL: activities of daily living
EMG: electromyography
3D: three-dimensional
MNI: Montreal Neurological Institute
