# Supplementary material for: Cerebral blood perfusion changes in amputees with myoelectric hands after rehabilitation: a SPECT computer-aided analysis
Source: BMC Neurosci. 2016 Aug 31;17(1):59. doi: 10.1186/s12868-016-0294-3 (PMC5006566; doi:10.1186/s12868-016-0294-3)
Supplement: Supplementary file 1 — 10.1186/s12868-016-0294-3 The contents of Brief Fugl-Meyer Scale and ADL Assessment. [file 12868_2016_294_MOESM1_ESM.docx]

**Additional file 1, The contents of Brief Fugl-Meyer Scale and ADL Assessment**

**Brief Fugl-Meyer Scale**

**I. Reflex activity**

- Attempt to elicit the biceps and triceps reflexes.
- Scoring (Maximum possible score = 4):
- No reflex activity can be elicited
- (2) - Reflex activity can be elicited

**II. Flexor synergy**

- Subject is sitting.
- The shoulder should be abducted at least 90 degrees. The starting position should be that of full extensor synergy. If the patient cannot actively achieve the starting position, the limb may be passively placed extended towards opposite knee in shoulder adduction/internal rotation and forearm pronation.

• Items to be scored are: Elevation (scapular), shoulder retraction (scapular), shoulder abduction (at least 90 degrees) and external, forearm supination

• Scoring (Maximum possible score = 10):

- - Cannot be performed at all
- - Performed partly

• (2) - Performed faultlessly Items to be scored are:

**III. Extensor synergy**

• Subject is sitting.

• Instruct the patient to adduct & internally rotate the shoulder, extend his arm towards the unaffected knee with the forearm pronated.

• The starting position should be that the limb is passively placed at patient’s side in elbow flexion and supination. The examiner must ensure that the patient does not rotate and flex the trunk forward, thereby allowing gravity to assist with the movement. The pectoralis major and triceps brachii tendons may be palpated to assess active movement.

- Items to be scored are: Shoulder adduction/internal rotation and forearm pronation.

• Scoring (Maximum possible score = 4):

• (0) - Cannot be performed at all

• (1) - Performed partly

• (2) - Performed faultlessly

**V. Movement combining synergies (4a, 4b, 4c)**

The patient is asked to perform three separate movements:

4a. Hand to lumbar spine:

• Subject is sitting with hand resting on lap.

• Subject is instructed to actively position the affected hand on the lumbar spine by asking them to “put your hand behind your back”.

• Scoring (Maximum possible score = 2):

• (0) – No specific action is performed (or patient moves but does not reach ASIS)

• (1) - Hand must pass anterior superior iliac spine (performed partly)

• (2) - Performed faultlessly (patient clears ASIS and can extend arm behind back towards sacrum)

4b. Shoulder flexion to 90°, elbow at 0°

• Subject is sitting with hand resting on lap.

• On the affected side, check subject’s available

PROM for shoulder flexion to 90° and full elbow extension.

• Subject is instructed to flex the shoulder to 90°, keeping the elbow extended. The elbow must be fully extended throughout the shoulder flexor movement; the forearm can be in pronation or in a mid-position

- Scoring (Maximum possible score = 2):

• (0) – Arm is immediately abducted, or elbow flexes at start of motion

• (1) - Abduction or elbow flexion occurs in later phase of motion

• (2) - Performed faultlessly (patient can flex shoulder keeping elbow extended)

**VI. Normal Reflexes (sitting)**

• The examiner shall elicit biceps and triceps phasic reflexes with a reflex hammer and finger flexors with quick stretch to the affected arm and note if the reflexes are hyperactive or not.

• Scoring (Maximum possible score = 2):

• (0) - At least 2 of the 3 phasic reflexes are markedly hyperactive

• (1) – One reflex is markedly hyperactive or at least 2 reflexes are lively

• (2) - No more than one reflex is lively, and none are hyperactive

**VII. Hand (8a, 8b, 8c, 8d, 8e, 8f)**

Finger mass flexion:

• Subject is sitting with arm on bedside table or lap.

• Starting from the position of finger extension (this may be attained passively if necessary), instruct the patient to fully flex all fingers.

• Scoring (Maximum possible score = 2):

• (0) – No flexion occurs

• (1) – Some flexion, but not full motion

• (2) – Completed active flexion

Grasp I

- Subject is sitting with arm on bedside table.

• Instruct the patient to abduct the thumb to grasp a piece of paper (tester may insert the paper). Then ask the patient to perform pure thumb adduction with the scrap of paper interposed between the thumb and first digit (as in figure). Test this grip against resistance by asking the patient to hold as you attempt to pull the paper out with a slight tug.

• Scoring (Maximum possible score = 2)

• (0) – Function cannot be performed

• (1) – Scrap of paper interposed between the thumb and index finger can be kept in place, but not against a slight tug

- (2) – Paper is held firmly against a tug

Grasp II

- Subject is sitting with arm on bedside table.

• Instruct the patient to grasp a pen (preferred –pen with cap) by opposing the thumb and index finger pads around the pen. The tester may support the patient’s arm but may not assist with the hand function required for the retrieval task. The pen may not be stabilized by the therapist or the patient’s other hand. To minimize excessive movement, however, a pen with a ‘pocket clip’ that prevents rolling more than 180° may be used.

- Once the pencil is retrieved, instruct the patient to oppose the thumb pad against the pad of the index finger with a pencil interposed. Test this grip against resistance by asking the patient to hold as you attempt to pull the pencil out with a slight tug.

• Scoring (Maximum possible score = 2)

• (0) – Function cannot be performed

• (1) – A pencil interposed between the thumb pad and the pad of the index finger can be kept in place, but not against a slight tug

- (2) – Pencil is held firmly against a tug

Grasp III

- Subject is sitting with arm on bedside table.

• Instruct the patient to grasp a small can (placed upright on a table without stabilization) by opening the fingers and opposing the volar surfaces of the thumb and digits. The arm may be supported but the tester may not assist with hand function.

• Once the can is grasped, test this grip against resistance by asking the patient to hold as you attempt to pull the can out with a slight tug.

• Scoring (Maximum possible score = 2)

• (0) – Function cannot be performed

• (1) – A can interposed between the thumb and index finger can be kept in place, but not against a slight tug

- (2) – Can is held firmly against a tug

Grasp IV

- Subject is sitting with arm on bedside table.

• Instruct the patient to perform a spherical grasp by grasping a tennis ball The tester may support the patient’s arm but may not assist with the hand function required for the retrieval task. The ball may not be stabilized by the therapist or the patient’s other hand. To minimize excessive movement, the ball can be placed on an object that reduces rolling. An inverted medium-sized bottle cap or other small ‘bowl’ shaped object that fits under the ball to prevent rolling is acceptable. (A Snapple-type bottle’s cap works well).Once the tennis ball is grasped, test this grip against resistance by asking the patient to hold as you attempt to pull the ball out with a slight tug.

• Scoring (Maximum possible score = 2)

• (0) – Function cannot be performed

• (1) – A tennis ball can be kept in place with a spherical grasp, but not against a slight tug

- (2) – Tennis ball is held firmly against a tug

**ADL Assessment**

| **Subject** | **0(Poor)** | **1(Good)** | **2(Excellent)** |
| --- | --- | --- | --- |
| Grab glass |  |  |  |
| Move glass |  |  |  |
| Hold heavy weights |  |  |  |
| Turn on paper |  |  |  |
| Open the door |  |  |  |
| Hold the glass and open it |  |  |  |
| Fetch heavy weights |  |  |  |
| Continuity |  |  |  |
