# Supplementary material for: Cerebral blood perfusion changes in amputees with myoelectric hands after rehabilitation: a SPECT computer-aided analysis
Source: BMC Neurosci. 2016 Aug 31;17(1):59. doi: 10.1186/s12868-016-0294-3 (PMC5006566; doi:10.1186/s12868-016-0294-3)
Supplement: Supplementary file 2 — 10.1186/s12868-016-0294-3 The change-rate of the 5 participants. [file 12868_2016_294_MOESM2_ESM.docx]

**Additional file 2, The change-rate of the 5 participants**

**Participant 1**

**Change-rate: mean±SD:** 29.20 ± 0.8059, N=7

| **Cluster No** | **Mean** | **Median** | **Max** | **Min** | **Pixel_Num** |
| --- | --- | --- | --- | --- | --- |
| 1 | 30.7 | 27.5 | 88.9 | 20.5 | 5024 |
| 2 | 26.6 | 24.7 | 44.4 | 20.8 | 202 |
| **3** | **31.0** | **29.2** | **84.7** | **20.5** | **1140** |
| 4 | 25.7 | 25.0 | 40.3 | 20.8 | 531 |
| 5 | 30.3 | 28.4 | 53.5 | 20.5 | 208 |
| 6 | 30.3 | 28.4 | 53.8 | 20.5 | 455 |
| **7** | **29.8** | **27.0** | **72.2** | **20.5** | **869** |

**Participant 2**

**Change-rate: mean±SD:** 27.90 ± 0.6423, N=9

| **Cluster No** | **Mean** | **Median** | **Max** | **Min** | **Pixel_Num** |
| --- | --- | --- | --- | --- | --- |
| 1 | 29.3 | 28.0 | 50.7 | 20.0 | 220 |
| 2 | 29.5 | 26.4 | 52.6 | 20.0 | 445 |
| 3 | 25.1 | 24.6 | 42.1 | 20.0 | 293 |
| 4 | 29.3 | 26.4 | 58.5 | 20.0 | 893 |
| 5 | 26.9 | 26.0 | 42.1 | 20.8 | 225 |
| 6 | 28.1 | 26.0 | 50.0 | 20.0 | 435 |
| 7 | 25.1 | 24.7 | 37.2 | 20.0 | 380 |
| 8 | 27.4 | 25.0 | 54.2 | 20.8 | 451 |
| 9 | 30.4 | 26.4 | 65.1 | 20.0 | 558 |

**Participant 3**

**Change-rate: mean±SD:** 30.20 ± 1.234, N=4

| **Cluster No** | **Mean** | **Median** | **Max** | **Min** | **Pixel_Num** |
| --- | --- | --- | --- | --- | --- |
| 1 | 28.7 | 25.9 | 62.5 | 20.3 | 493 |
| 2 | 30.4 | 26.9 | 73.8 | 20.4 | 447 |
| 3 | 28.1 | 25.9 | 53.8 | 20.3 | 325 |
| 4 | 33.6 | 29.6 | 74.1 | 20.3 | 477 |

**Participant 4**

**Change-rate: mean±SD:** 26.20 ± 0.4416, N=4

| **Cluster No** | **Mean** | **Median** | **Max** | **Min** | **Pixel_Num** |
| --- | --- | --- | --- | --- | --- |
| 1 | 27.4 | 26.3 | 44.0 | 20.2 | 512 |
| 2 | 26.3 | 24.2 | 48.2 | 20.2 | 342 |
| 3 | 25.7 | 23.0 | 50.0 | 20.0 | 1371 |
| 4 | 25.4 | 24.2 | 43.5 | 20.2 | 212 |

**Participant 5**

**Change-rate: mean±SD:**26.53 ± 0.4732, N=4

| **Cluster No** | **Mean** | **Median** | **Max** | **Min** | **Pixel_Num** |
| --- | --- | --- | --- | --- | --- |
| 1 | 26.1 | 24.5 | 44.3 | 20.1 | 419 |
| 2 | 27.7 | 26.4 | 46.4 | 20.5 | 275 |
| 3 | 25.5 | 23.2 | 39.5 | 20.5 | 298 |
| 4 | 26.8 | 26.2 | 41.1 | 20.5 | 257 |
