# Supplementary figures and images for: Cerebral blood perfusion changes in amputees with myoelectric hands after rehabilitation: a SPECT computer-aided analysis
Source: BMC Neurosci. 2016 Aug 31;17(1):59. doi: 10.1186/s12868-016-0294-3 (PMC5006566; doi:10.1186/s12868-016-0294-3)

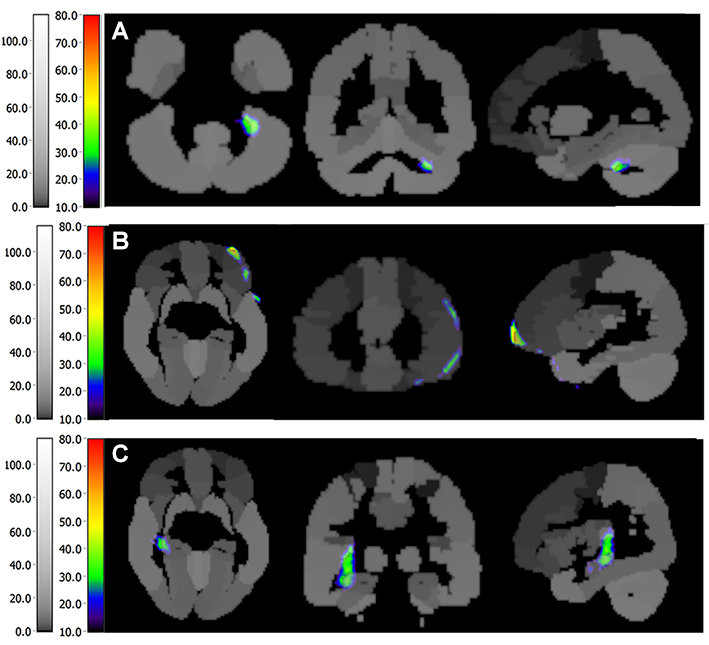

Supplement: Supplementary file 3 — 10.1186/s12868-016-0294-3 The activated regions of the five participants. [file 12868_2016_294_MOESM3_ESM.zip › S-p01.tif]

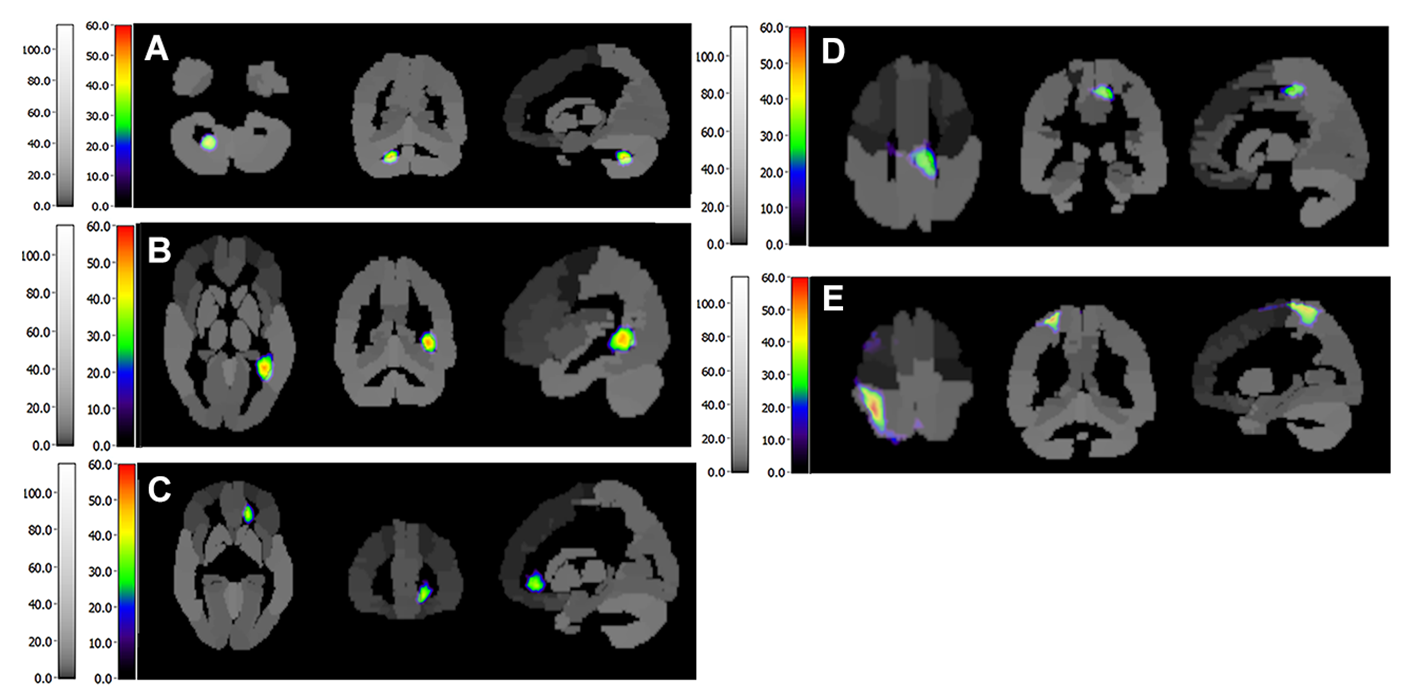

Supplement: Supplementary file 3 — 10.1186/s12868-016-0294-3 The activated regions of the five participants. [file 12868_2016_294_MOESM3_ESM.zip › s-p02.tif]

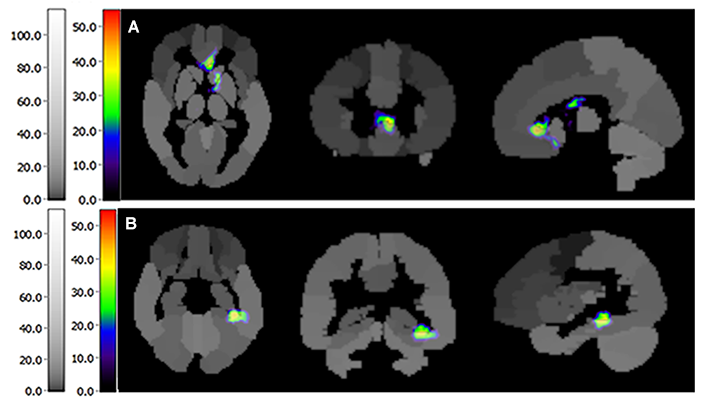

Supplement: Supplementary file 3 — 10.1186/s12868-016-0294-3 The activated regions of the five participants. [file 12868_2016_294_MOESM3_ESM.zip › s-p03.tif]

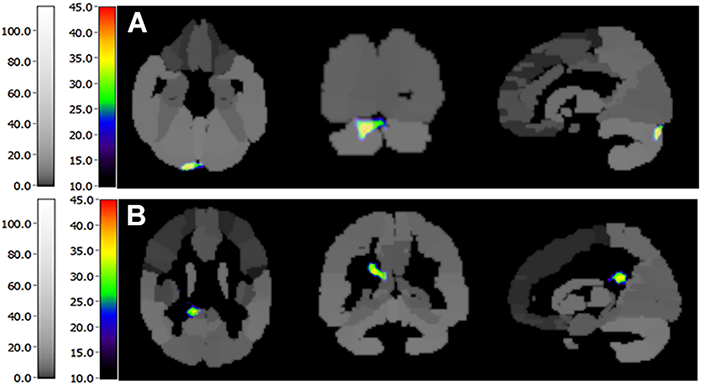

Supplement: Supplementary file 3 — 10.1186/s12868-016-0294-3 The activated regions of the five participants. [file 12868_2016_294_MOESM3_ESM.zip › s-p04.tif]

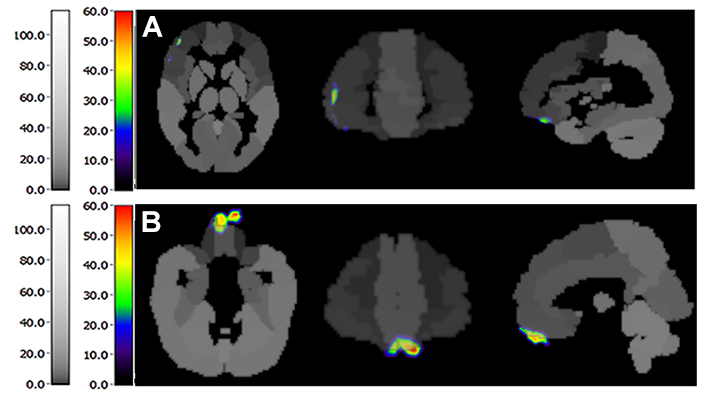

Supplement: Supplementary file 3 — 10.1186/s12868-016-0294-3 The activated regions of the five participants. [file 12868_2016_294_MOESM3_ESM.zip › s-p05.tif]
